# Intraoperative fluorescence in solid head and neck cancer: A scoping review

**DOI:** 10.1007/s00405-025-09442-5

**Published:** 2025-05-17

**Authors:** Brian A. Keith, Alejandro R. Marrero-Gonzalez, Isabelle J. Chau, Shaun A. Nguyen, William G. Albergotti, Alexandra E. Kejner, Jason G. Newman

**Affiliations:** 1 Department of Otolaryngology-Head and Neck Surgery, Medical University of South Carolina, 135 Rutledge Avenue, MSC 550, Charleston, SC 29425 USA; 2 School of Osteopathic Medicine, Campbell University, Lillington, NC USA; 3 The University of Puerto Rico School of Medicine, San Juan, Puerto Rico USA; 4 New York Medical College School of Medicine, Valhalla, NY USA

**Keywords:** Intraoperative imaging, Fluorescent-guided surgery, Intraoperative margin assessment, Head and neck cancer

## Abstract

**Purpose:**

Obtaining negative margins in primary tumor resection is essential to decreasing recurrence and mortality. Fluorescence imaging may aid in complete tumor removal. As fluorescent agents are still under clinical trial investigation for use in head and neck cancer (HNC), their effectiveness in intraoperative margin assessment (IMA) remains unclear. This scoping review examines the use of fluorescent-guided surgery (FGS) in the treatment of HNC, highlighting significant opportunities in this nascent field.

**Methods:**

PubMed, Scopus, CINAHL, and Cochrane Library were searched from inception through March 22, 2024. This study was conducted under PRISMA-ScR guidelines. Data on study characteristics, fluorescence and imaging techniques, imaging efficacy, and diagnostic accuracy were extracted.

**Results:**

Twenty-seven prospective studies from 2013 to 2024 on intraoperative FGS in HNC, involving 455 patients from six countries, were included. Studies ranged from preclinical to phase II trials, applying various fluorescent techniques, predominantly indocyanine green and IRDye800CW, to enhance surgical precision. Imaging assessments were conducted in-vivo, ex-vivo, or both, using a wide range of devices and taking an additional 0 to 30 min intraoperatively. Quantitative measures like signal-to-background ratio and mean fluorescent intensity suggested variable diagnostic accuracy across studies. FGS shows great potential in improving IMA, although standardization in methodologies and reporting is needed.

**Conclusion:**

This scoping review highlights the potential of intraoperative FGS to enhance treatment accuracy in solid HNC, though variability in diagnostic efficacy and a lack of standardized methodologies persist. Advancements in fluorophore technology and uniform procedural protocols are essential to optimize surgical outcomes and move towards personalized HNC interventions.

**Supplementary Information:**

The online version contains supplementary material available at 10.1007/s00405-025-09442-5.

## Introduction

Head and neck cancer (HNC) ranks among the top ten most common cancers globally, with approximately 950,000 new cases annually and a mortality-to-incidence ratio of 2 [1]. The primary treatment strategy for most solid HNC cases involves complete resection of the primary tumor. The goal of resection is to achieve negative margins on final pathology, which is associated with better survival, and decreased rates of local recurrence [2–4]. Despite these efforts, positive margin rates remain relatively high. In a 2018 National Cancer Database by Nocon et al., the rate of positive margins in head and neck squamous cell carcinoma was estimated to be 17.6% [5]. High margin rates may be partially attributed to inadequate visibility, poor tactile properties of the primary tumor, narrow margins between tumor and critical structures, which complicates an accurate assessment by the performing surgeon. Thus, there is a need to further explore and develop reliable intraoperative margin assessment (IMA) methods. Such novel techniques could enable surgeons to completely resect the primary tumor on the first attempt, reducing the need for additional procedures.

Frozen section analysis (FSA), originally introduced in the 1890 s, is a well-established method for IMA in HNC, though it has its limitations [6]. A recent meta-analysis reported that FSA’s sensitivity and specificity were 79.5% and 99.1%, respectively, suggesting that over 20% of positive margins may go undetected [7]. Additional challenges with FSA include the need for an available pathologist, additional costs, and extended duration of surgery [8].

Recently, fluorescent imaging has emerged as a promising alternative as it enhances tumor visualization and localization in real time through the use of fluorophores. Near-infrared fluorophores include indocyanine green (ICG) and IRDye800 CW [9, 10]. While ICG is non-specific, IRDye800 CW is often conjugated to tumor-specific anti-epidermal growth factor receptor (anti-EGFR) antibodies, such as panitumumab or cetuximab. Other fluorescent agents include 5-aminolevulinic acid (5-ALA), ONM-100 (now pegsitacianine), and cyanine-5 (Cy5).

Although various fluorophores have shown potential in IMA, only ICG, 5-ALA, and fluorescein have been approved by the Food and Drug Administration (FDA), and no fluorophores have been licensed for clinical use in HNC. To date, the use of fluorophores in HNC has been limited to phase II clinical trials. Moreover, establishing an optimal technique is challenging due to extensive methodological heterogeneity and underreporting of imaging efficacy and diagnostic data in the available literature [7, 11]. This scoping review aims to explore prospective studies on fluorophores in intraoperative fluorescent-guided surgery (FGS) for solid HNC and to identify gaps preventing a consensus on the best practices.

## Materials and methods

This scoping review was conducted per the Preferred Reporting Items for Systematic Reviews and Meta-Analyses extension for Scoping Reviews (PRISMA-ScR) guidelines [12]. This scoping review was guided by the Arksey and O’Malley five-stage methodological framework [13]. No systematic or scoping reviews for this topic were identified in peer-reviewed publications. No protocols on the topic were identified in databases of prospectively registered reviews (e.g. Prospero).

### Stage 1: Identify the research question

This review sought to investigate the use of intraoperative fluorescence in prospective studies for tumor margin assessment in all types of solid HNC. We aimed to assess the availability of imaging efficacy data and diagnostic metric data as well as the heterogeneity of intraoperative fluorescent techniques utilized among prospective studies.

### Stage 2: Identify relevant literature

The search strategy was designed and executed by two authors (BAK and ARMG) (Online Appendix 1). A systematic review was conducted with PubMed (US National Library of Medicine, National Institutes of Health), SCOPUS (Elsevier), CINAHL (EBSCO), and Cochrane Library (Wiley) databases from the date of inception to March 22nd, 2024, using keywords related to intraoperative imaging techniques, fluorophores, mean fluorescent intensity (MFI), tumor-to-background ratio (TBR), signal-to-background ratio (SBR), and HNC. The search aimed to identify all English articles related to intraoperative fluorescent imaging in HNC. The initial search was designed for PubMed and utilized medical subject headings [MeSH], title and abstract [tiab], and textword [tw]. The search was adapted for the other databases (SCOPUS, CINAHL, and Cochrane) by replacing MeSH terms with similar keywords. All articles from the search were exported to Covidence (Veritas Health Innovation Ltd., Melbourne, Australia), a review management software, for screening. All identified citations were collated and uploaded into EndNote (Clarivate Analytics, Philadelphia, PA, USA).

### Stage 3: Study selection

Two authors (BAK and ARMG) independently screened texts by title and abstract, then by full text using Covidence. Conflicts were resolved by two authors (BAK and ARMG) with the final resolution involving a third author (SAN) when necessary. The inclusion criteria required included articles to be full-text, primary studies, English-language, and published in a peer-reviewed journal. This scoping review considered randomized clinical trials, non-randomized clinical trials, and pilot studies. Exclusion criteria included retrospective cohort studies, chart reviews, case–control studies, case series, case reports, reviews, study protocols, non-human studies, and non-English language. Studies were only included if they utilized fluorescent imaging to assess confirmed HNCs intraoperatively.

#### Search process

**Fig. 1:**
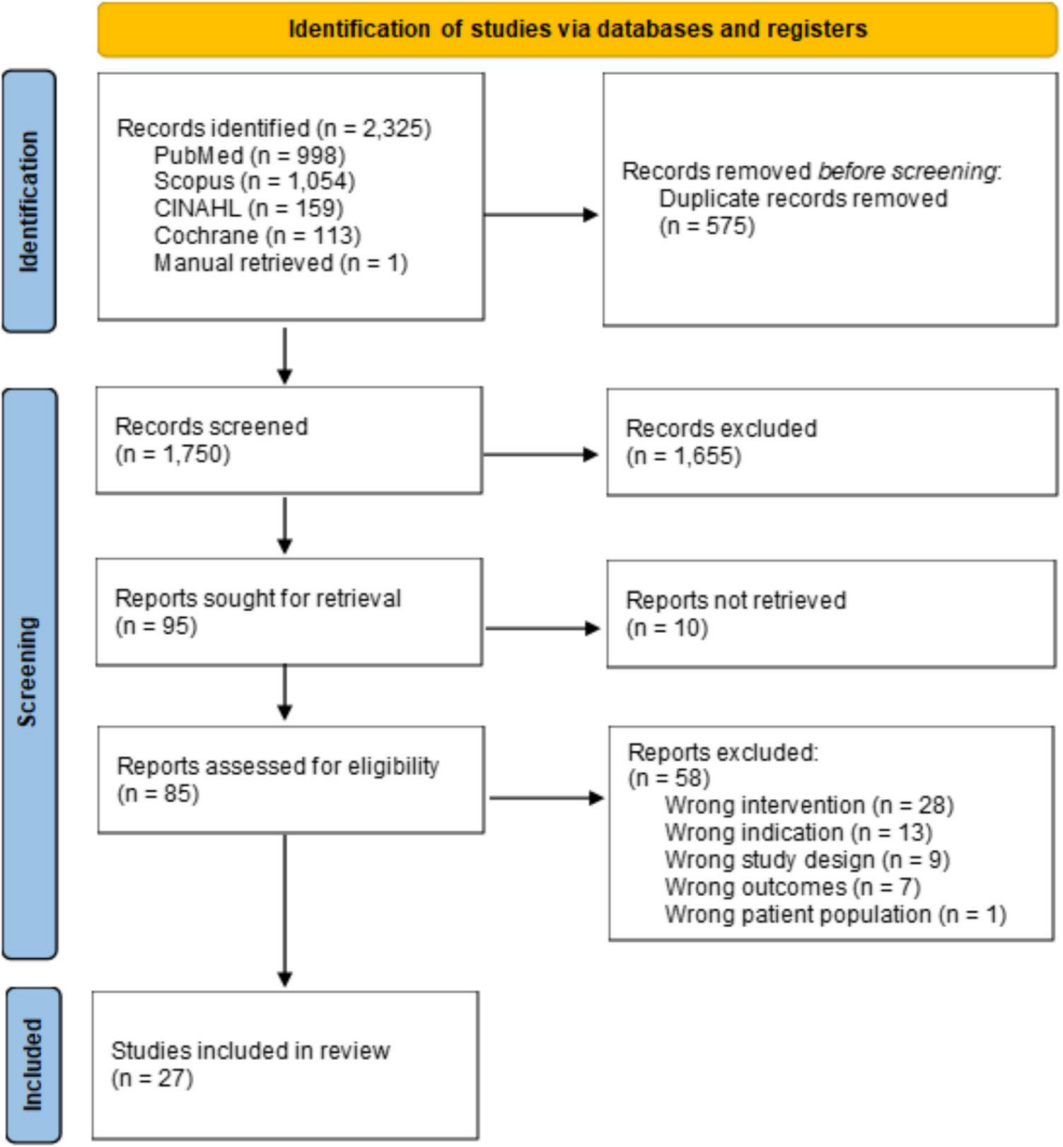
PRISMA 2020 flow diagram for new systematic reviews which included searches of databases and registers only

This scoping review was conducted in compliance with PRISMA-ScR scoping review methods. The initial search yielded 2,325 articles with 575 duplicate articles removed. After duplicates were removed, 1,750 articles remained. The titles and abstracts of the remaining articles were screened for relevance by two independent reviewers (BAK and ARMG). Conflicts were resolved by discussion between the two independent reviewers or with a third reviewer (SAN). Ten studies did not have full text to review. A total of 85 articles were assessed for eligibility, with 58 articles excluded at the full-text stage. Reasons for exclusion included wrong intervention, wrong indication, wrong study design, wrong outcomes, and wrong patient population. Specific exclusion criteria included studies that used fluorescence solely for lymph node identification, relied on autofluorescence, focused on pre-operative or post-operative fluorescent imaging, or employed visual dyes lacking fluorescent properties. After the search, 27 studies were deemed eligible for inclusion. The PRISMA flow diagram is shown in Fig. 1 and the PRISMA-ScR checklist is presented in Online Appendix 2.

### Stage 4: Charting the data

#### Data extraction

Data extraction for the 27 included studies was performed independently by two reviewers (BAK and ARMG). Study characteristics were collected, including author, year of publication, country of study, sample size, study design, and study objectives. Other data collected included patient demographics, imaging methods, fluorescent techniques, imaging efficacy data (MFI and SBR), and diagnostic accuracy measures (sensitivity, specificity, positive predictive value (PPV), and negative predictive value (NPV)).

#### Level of evidence and risk of bias

The level of evidence for included studies was assessed using the Oxford Center for Evidence-Based Medicine criteria [14]. Risk of bias was assessed using the Risk of Bias In Non-randomized Studies of Interventions (ROBINS-I) tool [15]. Risk of bias items included confounding, selection of participants into the study, classification of interventions, deviations from intended interventions, missing data, measurement of outcomes, and selection of reported results. Each item’s risk for bias was assigned a grade of low, unclear, or high. Assessment of Oxford level of evidence (OLE) and ROBINS-I was conducted independently by two authors (BAK and ARMG). Any conflicts were resolved by a third author (SAN).

### Stage 5: Collating, summarizing, and reporting results

**Fig. 2:**
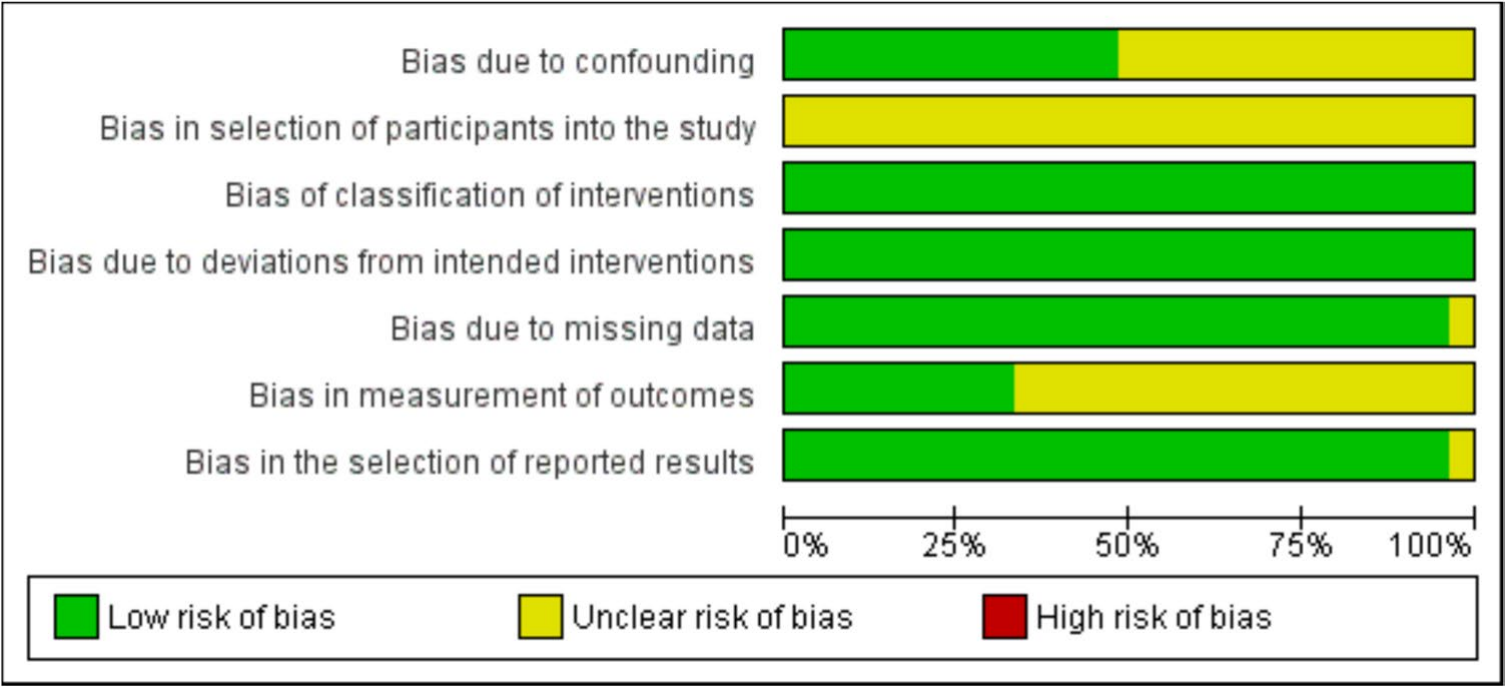
Risk of bias assessment for non-randomized studies of interventions

Relevant information from the included studies was narratively synthesized following guidance from the Cochrane Handbook for summarizing findings when meta-analysis was not feasible. Results of included studies and a narrative summary of our findings are presented through descriptive statistics (frequency (%), mean/median, and range/95% confidence interval (CI)) for categorical and continuous variables respectively. Risk of bias (Fig. 2, Online Appendix 3) figures were generated in Cochrane Review Manager (RevMan) version 5.4 (TheCochrane Collaboration 2020, United Kingdom). Qualitative data were synthesized in a narrative format. Implications of the analysis are then discussed for each fluorescent technique.

## Results

### Study characteristics

We included 27 studies published between 2013 and 2024 [16–42]. All included studies were conducted prospectively with 26 non-randomized studies. One study was an observational cohort study [22]. The studies were conducted globally with countries of origin including the United States (*n* = 15, 55.6%), Netherlands (*n* = 5, 18.5%), China (*n* = 3, 11.1%), Japan (*n* = 2, 7.4%), Germany (*n* = 1, 3.7%), and France (*n* = 1, 3.7%). Critical appraisal of included studies, shown in Fig. 2, demonstrates an overall acceptable risk of bias. The most significant sources of bias include confounding, selection of participants into the study, and measurement of outcomes. Appraisal of individual studies is shown in Online Appendix 3.

### Patient characteristics

The 27 studies included 455 patients with a mean age of 62.2 years (range: 2.1 to 90). The proportion of males-to-females was 55.0% (95% CI: 46.2% to 63.7%). Ethnicity was reported in only two studies with all patients identified as non-Hispanic whites [22, 32]. Across the 455 patients, 372 confirmed solid head and neck malignancies were identified with 367 squamous cell carcinomas, 1 malignant paraganglioma, 1 adenoid cystic carcinoma, 1 mucoepidermoid carcinoma, 1 epithelioid neoplasm with rhabdoid features, and 1 alveolar rhabdomyosarcoma. The majority of tumors were classified as T2, T3, and T4 at 39.5% (95% CI: 33.5% to 45.8%), 22.9% (95% CI: 15.1% to 31.8%), and 26.0% (95% CI: 20.5% to 32.1%), respectively. Specific tumor sites included in each study are shown in Online Appendix 4. Of the 372 confirmed malignancies, the most frequently reported tumor sites were within the oral cavity, including the tongue (*n* = 101, 27.1%), buccal mucosa (*n* = 26, 7.0%), and other oral cavity regions (*n* = 107, 28.8%), such as the floor of the mouth and gingiva. Less commonly reported sites were tonsils (*n* = 16, 4.3%), oropharynx (*n* = 8, 2.2%), nasal cavity (*n* = 5, 1.3%), parotid gland (*n* = 5, 1.3%), lip (*n* = 3, 0.8%), larynx (*n* = 2, 0.5%), and maxillary sinus (*n* = 2, 0.5%). The sites of remaining malignancies (*n* = 97, 26.1%) were not specified.

### Fluorescent techniques

Across the 27 studies, five fluorescent techniques were included: anti-EGFR monoclonal antibody tracers using IRDye800 CW (*n* = 13, 48%), ICG (*n* = 10, 37%), c-MET-targeted tracers using ICG or Cy5 (*n* = 2, 7.4%), ONM-100 (*n* = 1, 3.7%), and 5-ALA (*n* = 1, 3.7%). Fluorophore dosing protocols were included in 96.3% (26/27) of studies with 48.1% (13/27) studies implementing more than one dosing protocol. The time between administration of fluorophore and surgery/imaging was reported in 96.3% (26/27) of studies ranging from instantaneous to 7 days.

Among studies reporting specific tumor sites, the most commonly used techniques for each tumor site varied. Thirteen studies included tongue malignancies, using fluorescent techniques involving IRDye800 CW (*n* = 7, 53.8%), ICG (*n* = 5, 38.5%), and Cy5 (*n* = 1, 7.7%). Ten studies reported buccal tumors, using IRDye800 CW (*n* = 7, 70.0%) and ICG (*n* = 3, 30.0%). In the five studies reporting tumors of the oropharynx, IRDye800 CW (*n* = 3, 60.0%) and ICG (*n* = 2, 40.0%) were used. Two studies reporting lip tumors used ICG (*n* = 1, 50.0%) and IRDye800 CW (*n* = 1, 50.0%). All studies reporting tonsil data (*n* = 3, 100.0%) and parotid gland data (*n* = 2, 100.0%) used ICG. Studies reporting laryngeal tumors used ICG (*n* = 1, 50.0%) and 5-ALA (*n* = 1, 50.0%). Three studies with nasal cavity tumors used 5-ALA (*n* = 1, 33.3%) and IRDye800 CW (*n* = 2, 66.7%). Both studies with maxillary sinus data used IRDye800 CW (*n* = 2, 100.0%).

### Imaging techniques

Assessments of fluorescence were conducted in-vivo, ex-vivo, or both in 37% (10/27), 18.5% (5/27), and 44.4% (12/27) of studies, respectively. Additional time required before the case began and in the intraoperative period was explicitly discussed in 3.7% (1/27) and 44.4% (12/27) of studies, respectively. Extra time required during the intraoperative period was most frequently 5 min or less, ranging from 0 to 30 min.

The literature included both in-vivo and ex-vivo imaging approaches with a wide variety of devices, including Pearl-Trilogy® (*n* = 6; LI-COR BioSciences Inc., Lincoln, NE, USA), SurgVision Explorer Air® (*n* = 5; SurgVision, Groningen, the Netherlands), da Vinci Firefly™ Imaging System (*n* = 3; Intuitive Surgical, Sunnyvale, CA, USA), LUNA (*n* = 3; Novadaq Technologies, Inc., Mississauga, Ontario, Canada), SPY-PHI (*n* = 2; Novadaq Technologies, Inc., Mississauga, Ontario, Canada), IGP-ELVIS (*n* = 2; LI-COR Biosciences, Inc., Lincoln, NE, USA), REAL-IGS (*n* = 2; NuoYuan Medical Devices Co.,Ltd, Nanjing, China), Iridium (*n* = 2; VisionSense Corp, Philadelphia, Pennsylvania), HyperEye Medical System (*n* = 2; Mizuho Medical Co, Ltd, Tokyo, Japan), Karl Storz Endoskope with Cy5 filter (*n* = 1; Karl Storz, Tuttlingen, Germany), Artemis (*n* = 1; Quest Medical Imaging, Middenmeer, the Netherlands), blue light fluorescence-guided headlight system (*n* = 1; unspecified), PINPOINT (*n* = 1; Novadaq Technologies, Inc., Mississauga, Ontario, Canada), IMAGE1 S NIR/ICG system (*n* = 1; Karl Storz, Tuttlingen, Germany), HOPKINS® Telescope (*n* = 1; Karl Storz, Tuttlingen, Germany), SPY Elite® (*n* = 1; Stryker, Kalamazoo, MI, USA), Pearl® Imager (*n* = 1; LI-COR BioSciences Inc., Lincoln, NE, USA), and H2000 (*n* = 1; DPM, Beijing, China). Imaging systems utilized by each study are shown in Table 1.

**Table 1:** Study characteristics

Study	Patients (n)	OLE/Study Design	HNC Pathology	Extent of Tumor	Fluorophore	Dosing	Time from Study Drug to Imaging	Imaging System
[43]	10	4/PN	10 SCC	6 T1 4 T2	EMI-137 (c-MET-binding peptide-Cy5)	500 nmol	5 min	Karl Storz Endoskope with Cy5 filter (Karl Storz, Tuttlingen, Germany)
[44]	4	4/PN	4 SCC	2 T2 2 T4	ICG	25 mg/kg	30–45 min	Artemis (Quest Medical Imaging, Middenmeer, Netherlands)
[18]	16	3/PN	16 SCC	8 T1 4 T2 1 T3 3 T4	CTX-IRD	75 mg unlabeled CTX + 15 mg CTX-IRD	48 h	Explorer Air® (SurgVision, Groningen, the Netherlands) Pearl-Trilogy® (LI-COR BioSciences Inc., Lincoln, NE, USA)
[19]	65	2/PN	66 SCC	-	CTX-IRD	75 mg unlabeled CTX + 15 mg CTX-IRD	48 h	Explorer Air® (SurgVision, Groningen, the Netherlands) Pearl-Trilogy® (LI-COR BioSciences Inc., Lincoln, NE, USA)
[20]	7	4/PN	7 SCC	-	5-ALA	20 mg/kg	3–5 h	Blue light fluorescence-guided headlight system Operating microscope with blue light capabilities
[21]	21	2/PC	21 SCC	2 T0 3 T1 7 T2 5 T3 4 T4	PAN-IRD	0.06 mg/kg PAN-IRD (*n* = 3) 100 mg PAN + (0.05 mg/kg (*n* = 5) or 1 mg/kg (*n* = 7) PAN-IRD) 50 mg PAN-IRD (*n* = 6)	24–120 h	PINPOINT (Novadaq Technologies, Inc., Mississauga, Ontario, Canada) Explorer Air® (SurgVision, Groningen, the Netherlands) Pearl-Trilogy® (LI-COR BioSciences Inc., Lincoln, NE, USA)
[22]	18	4/PN	10 SCC	-	ICG	25 mg	6–8 h or 17–18 h	da Vinci Firefly™ Imaging System (Intuitive Surgical, Sunnyvale, CA, USA) Unspecified iv-vivo imaging system
[23]	18	2/PN	18 SCC	6 T2 4 T3 8 T4	PAN-IRD	50 mg	24–120 h	IGP-ELVIS (LI-COR Biosciences, Inc., Lincoln, NE, USA)
[24]	15	4/PC	15 SCC	-	CTX-IRD	2.5 mg/m^2^ (*n* = 3) 25 mg/m^2^ (*n* = 9) 62.5 mg/m^2^ (*n* = 3)	72–168 h	LUNA™ System (Novadaq Technologies, Inc., Mississauga, Ontario, Canada)
[25]	6	4/PC	6 SCC	-	CTX-IRD	(10 mg CTX (*n* = 3) or 100 mg CTX (*n* = 3)) + 25 mg/m^2^ CTX-IRD	72–96 h	LUNA™ System (Novadaq Technologies, Inc., Mississauga, Ontario, Canada)
[26]	24	2/PC	24 SCC	-	PAN-IRD	100 mg PAN + (0.5 mg/kg (*n* = 5) or 1 mg/kg (*n* = 7) PAN-IRD) 25 mg PAN-IRD (*n* = 6) 50 mg PAN-IRD (*n* = 6)	17–120 h	Pearl-Trilogy® (LI-COR BioSciences Inc., Lincoln, NE, USA) Unspecified in-vivo imaging system
[27]	20	4/PN	20 SCC	12 T2 5 T3 3 T4	ICG	0.75 mg/kg	6–8 h	REAL-IGS (n = 2; NuoYuan Medical Devices Co.,Ltd, Nanjing, China) with NIR spectrometer MAYA 2000 Pro (Ocean Optics, Dunedin, FL, USA)
[28]	8	4/PN	1 MEC 1 ENwRF 1 ARMS	1 T1 1 T2 1 T4	ICG	1.5 mg/kg	24 h	Iridium (VisionSense Corp, Philadelphia, Pennsylvania)
[29]	12	2/PC	12 SCC	1 T0 1 T1 6 T2 2 T3 2 T4	CTX-IRD	2.5 mg/m^2^ (*n* = 3) 25 mg/m^2^ (*n* = 6) 62.5 mg/m^2^ (*n* = 3)	72–96 h	LUNA™ System (Novadaq Technologies, Inc., Mississauga, Ontario, Canada) Pearl® Impulse (LI-COR BioSciences Inc., Lincoln, NE, USA)
[30]	55	4/PN	18 SCC	-	ICG	8.3 mg	0	IMAGE1 S NIR/ICG system (Karl Storz, Tuttlingen, Germany) with ICG HOPKINS telescope (Karl Storz, Tuttlingen, Germany)
[31]	6	4/PN	6 SCC	-	ICG	15 mg	0	da Vinci Firefly™ Imaging System (Intuitive Surgical, Sunnyvale, CA, USA)
[32]	14	4/PN	13 SCC 1 ACC	-	ICG	5 mg/kg	24 h	Iridium (VisionSense Corp, Philadelphia, Pennsylvania)
[33]	12	2/PC	12 SCC	2 T1 5 T2 4 T3 1 T4	PAN-IRD	25 mg (*n* = 4) or 50 mg (*n* = 8)	24–120 h	Pearl (LI-COR BioSciences Inc., Lincoln, NE, USA)
[34]	14	2/PN	11 SCC	5 T2 4 T3 2 T4	PAN-IRD	UNK	24–120 h	SPY-PHI (Novadaq Technologies, Inc., Mississauga, Ontario, Canada) Odyssey (LI-COR BioSciences Inc., Lincoln, NE, USA)
[35]	20	2/PC	18 SCC	1 T1 4 T2 5 T3 8 T4	PAN-IRD	25 mg or 50 mg	UNK	SPY-PHI (Novadaq Technologies, Inc., Mississauga, Ontario, Canada)
[36]	8	2/PC	6 SCC	4 T2 2 T3	PAN-IRD	25 mg or 50 mg	24–96 h	IGP-ELVIS (LI-COR Biosciences, Inc., Lincoln, NE, USA)
[37]	15	2/PC	15 SCC	-	CTX-IRD	10 mg CTX-IRD (*n* = 3) 25 mg CTX-IRD (*n* = 3) 50 mg CTX-IRD (*n* = 3) 75 mg CTX + (15 mg (*n* = 3) or 25 mg (*n* = 3) CTX-IRD)	96 h	Fluorescent imaging (SurgVision, Groningen, the Netherlands) attached to nasendoscope (Karl Storz, Tuttlingen, Germany) Explorer Air® (SurgVision, Groningen, the Netherlands) Pearl-Trilogy® (LI-COR BioSciences Inc., Lincoln, NE, USA)
[38]	30	2/PC	13 SCC	4 T1 3 T2 4 T3 2 T4	ONM-100	0.1 mg/kg (*n* = 3) 0.5 mg/kg (*n* = 2) 0.8 mg/kg (*n* = 1) 1.2 mg/kg (*n* = 7)	24 h (± 8 h)	Explorer Air® (SurgVision, Groningen, the Netherlands) Pearl-Trilogy® (LI-COR BioSciences Inc., Lincoln, NE, USA) SPY Elite® (Stryker, Kalamazoo, MI, USA) da Vinci Firefly™ Imaging System (Intuitive Surgical, Sunnyvale, CA, USA)
[40]	12	4/PC	12 SCC	9 T2 3 T4	ICG	0.5 mg/kg (*n* = 4) 0.75 mg/kg (*n* = 4) 1.0 mg/kg (*n* = 4)	30 min	REAL-IGS (NuoYuan Medical Devices Co.,Ltd, Nanjing, China) with NIR spectrometer MAYA 2000 Pro (Ocean Optics, Dunedin, FL, USA)
[39]	10	4/PC	9 SCC	-	c-MET-binding peptide-ICG	2.5 μmol (*n* = 5) 5.0 μmol (*n* = 5)	24 h	H2000 (DPM, Beijing, China)
[41]	9	4/PN	5 SCC	-	ICG	0.5 mg/kg	0–360 min	HyperEye Medical System (Mizuho Medical Co, Ltd, Tokyo, Japan)
[42]	6	4/PN	1 MP	1 T2	ICG	0.5 mg/kg	30–60 min	HyperEye Medical System (Mizuho Medical Co, Ltd, Tokyo, Japan)

*OLE* Oxford Level of Evidence; *PC* prospective cohort; *PN* prospective non-randomized; *ACC* adenoid cystic carcinoma; *ARMS* alveolar rhabdomyosarcoma; *ENwRF* epithelioid neoplasm with rhabdoid features; *MEC* mucoepidermoid carcinoma; *MP* malignant paraganglioma; *SCC* squamous cell carcinoma; *5-ALA* 5-aminolevulinic acid; *CTX* unlabeled cetuximab; *CTX-IRD* cetuximab-IRDye 800 CW; *ICG* indocyanine green; *PAN* unlabeled panitumumab; *PAN-IRD* panitumumab-IRDye 800 CW

### Imaging efficacy

Fifteen studies presented quantitative measures to assess the effectiveness of imaging, such as SBR or MFI [16, 17, 19, 21, 24–27, 29, 34, 35, 37–40]. Seven studies reported MFI, with metrics for solid tumors (6/7), adjacent normal tissue (4/7), wound beds (1/7), and peritumoral tissue (1/7). In terms of fluorescent techniques, MFI was documented in studies using ICG (2/7), IRDye800 CW (4/7), and c-MET-targeted tracers (1/7). SBR was detailed in 16 studies, using ICG (3/16), IRDye800 CW (10/16), c-MET-targeted tracers (2/16), and ONM-100 (1/16). MFI and SBR are presented in Table 2.

**Table 2:** Imaging efficacy and diagnostic accuracy of fluorescent techniques

Study	Setting	MFI	SBR		Sensitivity	Specificity
INDOCYANINE GREEN (Emission λ: 800–835 nm)						
[44]	In-vivo	Tumor Range: 52.3–103.6 AUs	Mean TBR: 1.37 (range: 0.879–2.12)	-		-
[22]	In-vivo & Ex-vivo	-	-		(Ex-vivo) Primary tumor: 100%	-
[27]	In-vivo	Primary Tumor: 398.863 ± 151.47 AUs Peritumoral: 278.52 ± 84.89 AUs Normal tissue: 274.5 ± 100.93 AUs	Mean TBR: 1.56 ± 0.41 Mean TPR: 1.45 ± 0.36		Primary tumor: 100% Residual/tumor bed: 100%	Residual/tumor bed: 88.9%
[28]	In-vivo & Ex-vivo	-	-		(In-vivo) Primary tumor: 100%	-
[30]	In-vivo	-	-		Primary tumor: 88.9%	-
[31]	In-vivo	-	-		Primary tumor: 0%	-
[32]	In-vivo	-	-		Primary tumor: 85.7%	-
[40]	In-vivo	-	Mean TBR: 0.5 mg/kg: 1.72 ± 0.03 1.0 mg/kg: 1.91 ± 0.36 0.75 mg/kg: 2.06 ± 0.23		-	-
[41]	In-vivo	-	-		-	-
[42]	In-vivo	-	-		-	-
ANTI-EGFR ANTIBODY TRACERS using IRDYE800 CW (Emission λ: ~ 820 nm)						
[18]	In-vivo & Ex-vivo	-	-		(Ex-vivo) Within 1 mm of tumor margin: 53.3% Within 5 mm of tumor margin: 96.7%	-
[19]	In-vivo & Ex-vivo	(In-vivo) Primary tumor: 3.3 (range: 2.7–6.1) × 10^−2^ mm^−1^	(In-vivo) Median TBR: 3.1 (range: 2.0–5.4)		(Ex-vivo) 100% at TBR ≥ 2 70.3% at TBR ≥ 1.5	(Ex-vivo) 85.9% at TBR ≥ 2 76.1% at TBR ≥ 1.5
[21]	In-vivo & Ex-vivo	-	(In-vivo) Mean TBR: 0.5 mg/kg: 2.4 ± 0.4 1 dd.0 mg/kg: 2.6 ± 0.4 50 mg: 2.5 ± 0.4 (Ex-vivo) Mean TBR: 0.06 mg/kg: 2.67 ± 0.7 0.5 mg/kg: 5.40 ± 0.6 1.0 mg/kg: 5.44 ± 0.7 50 mg: 6.53 ± 1.2		(In-vivo) 0.5 mg/kg: 100% for tumor-involved margin 50 mg: 100% for tumor-involved margin	(In-vivo) 0.5 mg/kg: 90% for tumor-involved margin 50 mg: 74% for tumor-involved margin
[23]	Ex-vivo	-	-		-	-
[24]	In-vivo & Ex-vivo	(3 representative patients) (In-vivo) Primary tumor: 20.6 ± 4.6 RFU Healthy tissue: 6.3 ± 0.5 RFU Post-resection wound bed: 2.0 ± 0.8 RFU	(In-vivo) TBR: 25 mg/m^2^ and 62.5 mg/m^2^ cohorts: range 2.2–14.1		-	-
[25]	In-vivo	-	Mean TBR: 100 mg: 5.5 ± 2.6 10 mg: 1.7 ± 0.7		-	-
[26]	In-vivo & Ex-vivo	(Ex-vivo) Primary tumor: 0.26 (? unit) Adjacent normal tissue: 0.09 (? unit)	(Ex-vivo) Mean TBR: 3.0 (range: 1.8–5.0)		-	-
[29]	In-vivo & Ex-vivo	-	(In-vivo) Mean TBR: 25 mg/m^2^: 4.3 (2.1–7.8) 62.5 mg/m^2^: 5.2 (4.8–6) (Ex-vivo) Mean TBR: 2.5 mg/m^2^: 5.9 25 mg/m^2^: 9.9 62.5 mg/m^2^: 5.7		-	-
[33]	Ex-vivo	-	-		Primary tumor: 100%	-
[34]	In-vivo & Ex-vivo	-	(In-vivo) TBR range: 1.8–2.7 Wound bed to normal tissue range: 0.2–0.7		(In-vivo) Primary tumor: 100%	-
[35]	In-vivo	-	Mean TBR: 2.2 ± 0.4		Primary tumor: 100%	-
[36]	Ex-vivo	-	-		95% at < 5 mm cut-off 95–100% at 2–5 mm cut-off 100% at < 2 mm cut-off	89% at < 5 mm cut-off 41–89% at 2–5 mm cut-off 1–41% at < 2 mm cut-off
[37]	In-vivo & Ex-vivo	-	(Ex-vivo) Mean TBR: 10 mg: 1.61 ± 0.93 25 mg: 2.02 ± 0.55 50 mg: 1.81 ± 0.32 75 mg + 15 mg: 3.06 ± 0.43 75 mg + 25 mg: 3.10 ± 2.53		(Ex-vivo) Resection margin: 100%	(Ex-vivo) Resection margin: 91%
c-MET-TARGETED TRACER using ICG (Emission λ: 800–835 nm) or CYANINE-5 (Emission λ: 670 nm)						
[43]	Ex-vivo	-	Median TBR: 2.50 (range 1.80–3.10)		90% for tumor-related fluorescence signal	-
[39]	In-vivo & Ex-vivo	(In-vivo) Primary tumor: 5.0-μM group: 47.35 ± 9.0 AUs 2.5-μM group: 32.45 ± 34.2 AUs Adjacent tissue: 5.0-μM group: 21.14 ± 8.2 AUs 2.5-μM group: 13.38 ± 15.2 AUs	(In-vivo) Mean TBR: 5.0-μM group: 2.71 ± 0.7 2.5-μM group: 3.11 ± 1.2		(Ex-vivo) 71% for tumor surface sample	(Ex-vivo) 83% for tumor surface sample
ONM-100 (Emission λ: 670 nm)						
[38]	In-vivo & Ex-vivo	-	(In-vivo) Median TBR: 1.2 mg/kg: 2.6 (IQR: 1.4)		(In-vivo) Resection margin: 100%	(In-vivo) Resection margin: 57%
5-AMINOLEVULINIC ACID (Emission λ: ~ 635 nm)						
[20]	In-vivo	-	-		85.8% for demonstration of fluorescence	-

*AU* arbitrary units; *MFI* mean fluorescent intensity; *RFU* relative fluorescent units; *SBR* signal-to-background ratio; *TBR* tumor-to-background ratio; *TPR* tumor-to-peritumoral ratio

### Diagnostic accuracy measures

Of the studies examined, 18 provided either explicit measures or sufficient data to calculate diagnostic statistics such as sensitivity, specificity, PPV, NPV, or accuracy [16, 18–22, 27, 28, 30–39]. Sensitivity and specificity were most frequently available in 18 and 7 studies, respectively. These are shown in Table 2.

## Discussion

Although FGS has been in use since the early 2000 s, clinical applications have developed as newer fluorescent markers and imaging devices have been introduced [45]. Our scoping review addresses significant gaps in the literature by summarizing prospective studies that use fluorescent tracers to intraoperatively assess solid HNC. Given the rising incidence of HNC, evaluating these emerging intraoperative fluorescent techniques and identifying shortcomings in the literature are important for guiding future research.

### Indocyanine green

ICG is the oldest and most studied fluorophore among the articles we identified. One recent systematic review published in 2022 assessed ICG’s diagnostic utility in the intraoperative assessment of HNCs which included 7 studies for ICG [11]. The authors concluded that heterogeneity in methodology among studies prevented a consensus on the utility of ICG. Despite identifying 5 additional studies for ICG in our scoping review, we found that the limitations described in their study continued to plague the literature. Age, race/ethnicity, and tumor characteristics are frequently unreported, which complicates meaningful comparisons between studies. This is especially problematic given the well-described significant influence these factors have on both the occurrence and the outcomes of HNC [46, 47].

Quantitative measures for imaging efficacy and diagnostic accuracy of ICG were reported in 8 studies, and the methods for assessing these outcomes were highly variable. The sensitivity of ICG for tumors is discussed in 5 studies, but typically only as it pertains to ICG positivity. Surprisingly, the sensitivity for margin assessment is reported in only one study. It would be beneficial for all future studies to identify the ability of intraoperative fluorescence to predict positive margins given its prognostic value [22]. This would allow for a more robust comparison between the various fluorescent techniques, as well as more established methods for intraoperative margin assessment such as FSA.

Although TBR, reported by 3 studies, is the most consistent measure for imaging efficacy across studies for ICG, the interpretation of its significance can only truly be appreciated in the context of fluorescence intensity. A study by de Wit et al. suggested the optimal cutoff for SBR was ≥ 2 for tumor-positive margin detection [19]. However, challenges such as limited fluorescence penetration in thicker tumors, insufficient fluorophore dosing, or delays between drug administration and surgery can lead to weak fluorescent intensity. Even with a TBR exceeding 2, these factors might hinder the surgeon's ability to distinguish between healthy and cancerous tissue due to inadequate signal intensity [19]. Therefore, the literature could be improved by future studies reporting MFI and SBR for all tissues studied to convey the value of ICG.

Most studies utilize ICG for in-vivo fluorescent imaging as opposed to the ex-vivo, or back table, approach, and both approaches can increase the time in the OR. The study by Cortese et al. reported an additional 30 min required for in-vivo analysis, they stated that this was due to necessary camera adjustments with the Artemis imaging system [17]. Unfortunately, no other studies utilized this imaging system so it is unclear whether the extended time would be necessary for all operators. No other ICG studies reported more than 5 additional minutes required during the intraoperative, suggesting that ICG is a time-efficient technique. Moving forward, we hope to see all future studies report the amount of time required before, during, and after the case to better understand the timing of ICG-guided surgery.

### Receptor-targeted IRDye800 CW

Epidermal growth factor receptor (EGFR), a tyrosine kinase receptor, is involved with cell differentiation and proliferation and is frequently overexpressed in cancer tissues. This overexpression presents a unique opportunity to target EGFR with FDA-approved monoclonal antibodies, such as cetuximab and panitumumab, conjugated to a fluorescent dye, IRDye800 CW [48]. Cetuximab-IRDye800 CW (CTX-IRD) and panitumumab-IRDye800 CW (PAN-IRD) are currently being studied in phase II trials for the intraoperative assessment of HNC. Historically, ICG has ruled the field of FGS despite their non-targeted approach, which can influence fluorescent signals due to vascular characteristics, necrosis, inflammation, and other factors [28]. In contrast, anti-EGFR tracers, which may be less likely to be influenced by the previously mentioned factors, have substantially risen in popularity over the past decade [7]. Perhaps this shift highlights the utility of a tumor-targeted, precision approach in intraoperative fluorescent surgery.

A recent meta-analysis analyzing the diagnostic accuracy of intraoperative techniques in HNC found the sensitivity and specificity of tumor-targeted fluorescence to be 95.7% and 82.7%, respectively [7]. Their meta-analysis included 4 anti-EGFR studies and 1 ONM-100 study. In our study, we identified 13 anti-EGFR studies in the literature with 3 studies overlapping with the previously mentioned meta-analysis. Four additional studies each included sensitivities of 100%, thus strengthening support for anti-EGFR tracers in the intraoperative management of HNC. However, as was the case with ICG, the methodology employed across studies was very heterogeneous. Timing between administration of the study drug and fluorescent imaging is one such example. Typically, patients receive anti-EGFR tracers between 1 and 5 days before imaging although it is unclear what effect this has on imaging efficacy and diagnostic accuracy; one study conducted imaging up to seven days after the study drug was given [24]. The early administration of anti-EGFR tracers is made possible by their longer half-life, which is 23–33 h, compared to other fluorophores [49]. In future studies, methods should be consistent with the published literature, and validated diagnostic measures should be included.

SBR and MFI are more frequently reported for this technique at 69.2% (9/13) and 13.0% (3/13) of studies, respectively. Despite the availability of these measures, the heterogeneity in dosing protocols presents significant challenges for meta-analysis. In two CTX-IRD dose-escalation studies, the LUNA™ system was used to explore weight-based cohorts at 2.5 mg/m^2^, 25 mg/m^2^, and 62.5 mg/m^2 ^[24, 29]. One study reported all in-vivo TBRs > 2 (range: 2.2 to 14.1) for the latter two cohorts, while the other reported in-vivo TBRs of 4.3 (range: 2.1–7.8) and 5.2 (range: 4.8–6). Interestingly, both studies reported no significant difference in in-vivo TBR between the middle and high-dose cohorts. The study by Rosenthal et al. concluded support of the 25 mg/m^2^ dose due to loss in optimal TBR observed at the highest dose [29]. Although no difference was observed, it is important to note that these findings developed from low-powered dose-escalation studies. In addition, while fluorescently tagged anti-EGFR antibodies have been in other studies to guide oncologic surgery in colon cancer, pancreatic cancer, and glioblastoma, these studies lack the power to determine optimal dosing for HNC studies. Future studies utilizing anti-EGFR should prioritize greater patient recruitment to enhance study power which could potentially reveal significant differences among the various dosing cohorts.

Across four anti-EGFR studies, there were 17 related adverse events reported with 16/17 being grade 2 [18, 21, 29, 37]. Bronchospasm, a grade 2 adverse event, was reported in one case with the cause being iatrogenic after the study fluorophore, CTX-IRD, was administered at a rate 10 × higher than recommended. Although no skin toxicities were reported for CTX-IRD and PAN-IRD in the included studies, we suspect this will change once studies progress to higher phases and recruit a greater number of patients. Additional fluorescent agents for future studies include nimotuzumab-IRDye800 CW (NIM-IRD). Nimotuzumab is a humanized monoclonal antibody with an intermediate affinity for EGFR that is used for inoperable HNC [50]. In a recent meta-analysis by Bernhard et al., nimotuzumab was found to have significantly fewer skin toxicities compared to cetuximab and panitumumab [51]. Switching to an anti-EGFR with a more favorable safety profile such as nimotuzumab once it is FDA-approved could be considered before advancing anti-EGFR FGS.

It should be noted that anti-EGFR antibody tracers, as well as other fluorophores, face inherent limitations due to the optical properties of biological tissues. Tissue scattering and absorption of light reduce the ability to detect fluorescence, making the assessment of deep margins particularly challenging [52]. Furthermore, imaging is often complicated by interference from ambient light and reflections. Ex-vivo, or back-table, imaging using closed-field systems can mitigate these issues by providing a more controlled environment [37, 53]. For example, Gao et al. demonstrated a TBR of 2.5 with in-vivo imaging compared to 5.8 using ex-vivo techniques, concluding that back-table imaging may offer more reliable and consistent data [21]. Future studies should explore comparisons between in-vivo imaging with open-field devices and ex-vivo imaging using closed-field systems to optimize surgical outcomes.

### c-MET-targeted tracers

Mesenchymal-epithelial transition factor (c-MET), the tyrosine receptor for hepatic growth factor (HGF), has been implicated in tumor cell proliferation and survival. Literature has reported enhanced c-MET expression and increased activity of the HGF/c-MET signaling pathway in HNC which suggests it may be an effective target for fluorescent tracers [54, 55].

We identified two prospective studies utilizing c-MET targeting tracers in the literature, comprising one cohort study and one non-randomized single-arm study, both with a study size of 10 patients [16, 39]. These studies used two different tracers. One employed a c-MET binding peptide conjugated to ICG, and the other used EMI-137, a peptide with a high affinity for c-MET conjugated to Cy5, a fluorescent dye visible in the far-red spectrum. Additionally, the two studies used distinct imaging systems to accommodate the different properties of the two tracers.

Despite consistent reporting of SBR and sensitivity for both studies, the inconsistencies in methodology pose challenges for comparisons between the two studies. Nonetheless, c-MET targeted tracers seem to reliably differentiate tumors from solid tissue with the median or mean TBR of both studies being ≥ 2. However, the sensitivity varied with one study reporting 90% and the other at 71% for detecting fluorescent activity in tumor tissue.

To assess the imaging and diagnostic capabilities of c-MET-targeted tracers more effectively, future studies should aim for more uniform methodology which would establish more definitive conclusions regarding their clinical utility.

### ONM-100

ONM-100 (now pegsitacianine) is a pH-sensitive polymer micelle that contains sequestered ICG, allowing for fluorescent activity to be observed in low-pH environments such as malignant tumors. One systematic review on IMA included 2 studies using ONM-100 in HNC [11]. The authors concluded that ONM-100 may be more sensitive for HNC, but less specific which was attributed to the smaller sample size of their included studies. In our study, we identified only one article using ONM-100 which was included in the systematic review [38]. The second article was excluded from our scoping review due to the retrospective analysis of fluorescent data. The included study comprised 13 HNCs and reported a sensitivity and specificity of 100% and 57%, respectively. In this dose escalation study, the authors were able to explore four different weight-based dosing regimens for ONM-100 and concluded that the 1.2 mg/kg cohort was optimal for tumor detection and sensitivity.

Results from a phase II study on intraoperative fluorescent imaging of lung cancer with ONM-100 (NCT05048082) were recently published [56, 57]. This study demonstrated that ONM-100 is well-tolerated, but that it did not consistently label malignant lung tissue, reporting a sensitivity of 31% and specificity of 33%. The authors suggested that other solid tumors may be more acidic than lung cancer, better activating this pH-sensitive nanoprobe. Regarding its utility in HNC, a phase II study evaluating its diagnostic performance in patients with unknown primary HNC is ongoing (NCT05576974) [58]. Little is known about ONM-100 given its recent conception, but its ability to selectively fluoresce in a tumor microenvironment seems promising.

### 5-Aminolevulinic acid

5-Aminolevulinic Acid (5-ALA) is a natural amino acid and a key intermediate in the heme synthesis pathway [59]. 5-ALA is metabolized to protoporphyrin IX (PpIX) in the mitochondria, and this metabolite accumulates in the mitochondria. When exogenous 5-ALA is administered, PpIX fluoresces when exposed to blue light and emits on the red-light spectrum [60]. Due to their high metabolic activity and increased permeability, cancer cells absorb and concentrate 5-ALA at higher levels compared to normal tissue. Given its favorable safety profile, 5-ALA has been FDA-approved for high-grade glioma resection and studied for intraoperative fluorescent guidance in many cancer types, including CNS, gastric, and breast [61–63].

In our study, we identified only one prospective study examining the use of 5-ALA in intraoperative management of solid HNC [20]. The study was appropriately low-powered (*N* = 7) given its distinction as a prospective pilot trial. The authors reported success with 85.8% sensitivity for robust fluorescence of the tumor and 100% sensitivity for detecting positive surgical margins. Of note, the one tumor that did not fluoresce was explained by the patient’s case by being delayed to 8.5 h after 5-ALA was given. Although blue light (405 nm) was used in this study, the authors did not specify specific equipment used for ex-vivo visualization. Subjective fluorescent intensity was used to report the fluorescence of the tumor. Therefore, future studies should report MFI and SBR to quantify fluorescence which would better describe the contrast of tumor tissue and normal tissue. Currently, the authors have advanced to a phase II clinical trial (NCT05101798), which is the only ongoing trial for 5-ALA in HNC we identified [64]. This will increase the power of the study, and therefore the diagnostic ability to determine tumor margins could be evaluated. The utility of 5-ALA could also be expanded by providing MFI and SBR.

In recent years, there has been growing interest in leveraging artificial intelligence (AI), machine learning, and deep learning to improve lesion and margin detection. Recent studies have demonstrated how AI can enhance the accuracy of tumor detection and classification to support clinicians in real-time decision-making [65, 66]. While this was not the focus of the present study, it is a rapidly expanding area of research that has shown promising results in tumor identification with the potential to further refine diagnosis and treatment planning.

### Limitations

There are certain limitations to the study design worth discussing. Primarily, the variability in the types of fluorophores used and the imaging techniques across the studies introduces significant heterogeneity, which complicates attempts to synthesize or compare outcomes directly. The absence of meta-analysis due to this heterogeneity restricts the quantitative assessment of the efficacy of fluorescent IGS techniques. Additionally, it is possible that the search strategy did not capture all relevant articles. Another limitation is the lack of treatment information for study patients. Previous radiotherapy could affect migration of fluorophores into cancerous tissue subsequently decreasing MFI of the site [67]. One paper included previously irradiated patients to understand how radiation could reduce ICG visibility [17]. Although the authors concluded that near-infrared imaging for ICG-guided surgery was still feasible, the results were hardly definitive as only four patients were included. There is no mention of chemotherapy affecting FGS in the literature. The effects of chemotherapy and radiotherapy on FGS are certainly topics worthy of future research. Due to our reliance on studies only published in English, we may have omitted international research potentially leading to bias in conclusions.

## Conclusions

This scoping review highlights the potential of intraoperative FGS in HNC treatment, despite current challenges in standardizing methodologies and reporting out-comes and the abundance of low-powered studies. The varying diagnostic efficacy of fluorophores like ICG and IRDye800 CW emphasizes the need for more studies with uniform protocols to advance this field and facilitate the licensing of these probes within HNC. Future studies should focus on refining these techniques to enhance the accuracy of margin assessments, reducing the necessity for subsequent surgeries, and improving patient outcomes. By developing and implementing advanced fluorophores and imaging systems, we can move closer to personalized surgical interventions in HNC, potentially transforming treatment paradigms.

## Supplementary Information

Below is the link to the electronic supplementary material.

Supplementary file1 (172 KB)

## Data Availability

All data will be made available upon request.
