# Supplementary material for: Intraoperative fluorescence in solid head and neck cancer: A scoping review
Source: Eur Arch Otorhinolaryngol. 2025 May 17;282(11):5469–84. doi: 10.1007/s00405-025-09442-5 (PMC12605510; doi:10.1007/s00405-025-09442-5)
Supplement: Supplementary file 1 — Supplementary file1 (172 KB) [file 405_2025_9442_MOESM1_ESM.docx]

**Online Appendix 1:** *Search Strategy*

PubMed

(("Head and Neck Neoplasms"[Mesh] OR "head neoplasm"[tw] OR "neck neoplasm"[tw] OR

"oropharyngeal cancer"[tw] OR "Hypopharyngeal cancer"[tw] OR "squamous cell

carcinoma"[tw] OR "laryngeal cancer"[tw] OR "lip cancer"[tw] OR "oral cavity cancer"[tw] OR

"salivary gland cancer"[tw] OR "nasopharyngeal cancer"[tw] OR "paranasal sinus cancer"[tw]

OR "salivary gland cancer"[tw] OR "nasal cavity cancer"[tw] OR "head and neck

carcinoma"[tw] OR "head and neck squamous cell carcinoma"[tw]) AND ("Fluorescence"[tiab]

OR "fluorescence imaging"[tw] OR "fluorescent"[tw] OR "fluorescence-guided"[tw] OR

"fluorescence visualization"[tw] OR "image-guided"[tw] OR "intraoperative"[tiab] OR "back-

table"[tw] OR "real-time"[tw] OR "navigation"[tw] OR "imaging"[tw] OR "near-infrared"[tw]

OR "NIR"[tw] OR "fluobeam"[tw] OR "excitation laser"[tw] OR "bluelight"[tw] OR

"VELscope"[tw] OR "Spy-phi"[tw] OR "LUNA" [tw] OR "FLIM"[tw] OR "in situ imaging"[tw]

OR "ex vivo imaging"[tw]) AND ("Fluorescent Dyes"[Mesh] OR "Indocyanine green"[tw] OR

"Cy5 Dye"[tw] OR "Cyanine 5 dye"[tw] OR "Cetuximab"[tw] OR "panitumumab"[tw] OR "anti-

EGFR"[tw] OR "EGFR-targeted"[tw] OR "IRDye800CW"[tw] OR "antibody fluorescence"[tw]

OR "fluorescently-labeled antibody" OR "hybrid nanocolloid"[tw] OR "autofluorescence"[tw]

OR "5-aminolevulinic acid"[tw] OR "c-MET"[tw] OR "nanoparticles"[tw] OR "hybrid

tracer"[tw] or "MFI"[tw] OR "mean fluorescence intensity"[tw] or "margin assessment"[tw])

AND ("trial"[tw] or "clinical trial"[tw] or "pilot study"[tw] or "pilot trial"[tw] OR

"prospective"[tw] or "randomized"[tw] or "control"[tw] or "intervention"[tw] or "feasibility"[tw]

or "proof of concept"[tw])) OR ("Head and Neck Neoplasms"[Mesh] AND (Fluorescen* or

intraoperative) AND (clinicaltrial[Filter]))

998 results

CINAHL

(("Head and Neck Neoplasms" OR "head neoplasm" OR "neck neoplasm" OR "oropharyngeal

cancer" OR "Hypopharyngeal cancer" OR "squamous cell carcinoma" OR "laryngeal cancer" OR

"lip cancer" OR "oral cavity cancer" OR "salivary gland cancer" OR "nasopharyngeal cancer"

OR "paranasal sinus cancer" OR "salivary gland cancer" OR "nasal cavity cancer" OR "head and

neck carcinoma" OR "head and neck squamous cell carcinoma") AND ("Fluorescence" OR

"fluorescence imaging" OR "fluorescent" OR "fluorescence-guided" OR "fluorescence

visualization" OR "image-guided" OR "intraoperative" OR "back-table" OR "real-time" OR

"navigation" OR "imaging" OR "near-infrared" OR "NIR" OR "fluobeam" OR "excitation laser"

OR "bluelight" OR "VELscope" OR "Spy-phi" OR "LUNA" OR "FLIM" OR "in situ imaging"

OR "ex vivo imaging") AND ("Fluorescent Dyes" OR "Indocyanine green" OR "Cy5 Dye" OR

"Cyanine 5 dye" OR "Cetuximab" OR "panitumumab" OR "anti-EGFR" OR "EGFR-targeted"

OR "IRDye800CW" OR "antibody fluorescence" OR "fluorescently-labeled antibody" OR

"hybrid nanocolloid" OR "autofluorescence" OR "5-aminolevulinic acid" OR "c-MET" OR

"nanoparticles" OR "hybrid tracer" or "MFI" OR "mean fluorescence intensity" or "margin

assessment") AND ("trial" or "clinical trial" or "pilot study" or "pilot trial" OR "prospective" or

"randomized" or "control" or "intervention" or "feasibility" or "proof of concept")) OR (( head

and neck cancer or oral cancer or oropharyngeal cancer or hnc or head and neck neoplasms )

AND (fluorescence or intraoperative) AND (trial))

159 results

COCHRANE

(("Head and Neck Neoplasms" OR "head neoplasm" OR "neck neoplasm" OR "oropharyngeal

cancer" OR "Hypopharyngeal cancer" OR "squamous cell carcinoma" OR "laryngeal cancer" OR

"lip cancer" OR "oral cavity cancer" OR "salivary gland cancer" OR "nasopharyngeal cancer"

OR "paranasal sinus cancer" OR "salivary gland cancer" OR "nasal cavity cancer" OR "head and

neck carcinoma" OR "head and neck squamous cell carcinoma") AND ("Fluorescence" OR

"fluorescence imaging" OR "fluorescent" OR "fluorescence-guided" OR "fluorescence

visualization" OR "image-guided" OR "intraoperative" OR "back-table" OR "real-time" OR

"navigation" OR "imaging" OR "near-infrared" OR "NIR" OR "fluobeam" OR "excitation laser"

OR "bluelight" OR "VELscope" OR "Spy-phi" OR "LUNA" OR "FLIM" OR "in situ imaging"

OR "ex vivo imaging") AND ("Fluorescent Dyes" OR "Indocyanine green" OR "Cy5 Dye" OR

"Cyanine 5 dye" OR "Cetuximab" OR "panitumumab" OR "anti-EGFR" OR "EGFR-targeted"

OR "IRDye800CW" OR "antibody fluorescence" OR "fluorescently-labeled antibody" OR

"hybrid nanocolloid" OR "autofluorescence" OR "5-aminolevulinic acid" OR "c-MET" OR

"nanoparticles" OR "hybrid tracer" or "MFI" OR "mean fluorescence intensity" or "margin

assessment") AND ("trial" or "clinical trial" or "pilot study" or "pilot trial" OR "prospective" or

"randomized" or "control" or "intervention" or "feasibility" or "proof of concept")) OR ((head

and neck cancer):ti,ab,kw AND (fluorescence):ti,ab,kw)

113 results

Scopus

((TITLE-ABS-KEY("Head and Neck Neoplasms" OR "head neoplasm" OR "neck neoplasm"

OR "oropharyngeal cancer" OR "Hypopharyngeal cancer" OR "laryngeal cancer" OR "lip

cancer" OR "oral cavity cancer" OR "salivary gland cancer" OR "nasopharyngeal cancer" OR

"paranasal sinus cancer" OR "salivary gland cancer" OR "nasal cavity cancer" OR "head and

neck carcinoma" OR "head and neck squamous cell carcinoma")) AND (TITLE-ABS-

KEY("Fluorescence" OR "fluorescence imaging" OR "fluorescent" OR "fluorescence-guided"

OR "fluorescence visualization" OR "image-guided" OR "intraoperative" OR "back-table" OR

"real-time" OR "navigation" OR "imaging" OR "near-infrared" OR "NIR" OR "fluobeam" OR

"excitation laser" OR "bluelight" OR "VELscope" OR "Spy-phi" OR "LUNA" OR "FLIM" OR

"in situ imaging" OR "ex vivo imaging")) AND (TITLE-ABS-KEY("Fluorescent Dyes" OR

"Indocyanine green" OR "Cy5 Dye" OR "Cyanine 5 dye" OR "Cetuximab" OR "panitumumab"

OR "anti-EGFR" OR "EGFR-targeted" OR "IRDye800CW" OR "antibody fluorescence" OR

"fluorescently-labeled antibody" OR "hybrid nanocolloid" OR "autofluorescence" OR "5-

aminolevulinic acid" OR "c-MET" OR "nanoparticles" OR "hybrid tracer" or "MFI" OR "mean

fluorescence intensity" or "margin assessment")) AND (TITLE-ABS-KEY("trial" or "clinical

trial" or "pilot study" or "pilot trial" OR "prospective" or "randomized" or "control" or

"intervention" or "feasibility" or "proof of concept"))) OR (( TITLE-ABS-KEY ( head AND neck

AND cancer ) AND ( TITLE-ABS-KEY ( fluorescence ) OR TITLE-ABS-KEY ( intraoperative )

) AND TITLE-ABS-KEY ( clinical AND trial )))

1,054 results

Date Searched: March 22^nd^, 2024

**Online Appendix 2:** *Preferred Reporting Items for Systematic reviews and Meta-Analyses extension for Scoping Reviews (PRISMA-ScR) Checklist*

| **SECTION** | **ITEM** | **PRISMA-ScR CHECKLIST ITEM** | **REPORTED ON PAGE #** |
| --- | --- | --- | --- |
| **TITLE** | | | |
| Title | 1 | Identify the report as a scoping review. | 2 |
| **ABSTRACT** | | | |
| Structured summary | 2 | Provide a structured summary that includes (as applicable): background, objectives, eligibility criteria, sources of evidence, charting methods, results, and conclusions that relate to the review questions and objectives. | 2 |
| **INTRODUCTION** | | | |
| Rationale | 3 | Describe the rationale for the review in the context of what is already known. Explain why the review questions/objectives lend themselves to a scoping review approach. | 3 |
| Objectives | 4 | Provide an explicit statement of the questions and objectives being addressed with reference to their key elements (e.g., population or participants, concepts, and context) or other relevant key elements used to conceptualize the review questions and/or objectives. | 3 |
| **METHODS** | | | |
| Protocol and registration | 5 | Indicate whether a review protocol exists; state if and where it can be accessed (e.g., a Web address); and if available, provide registration information, including the registration number. | 4 |
| Eligibility criteria | 6 | Specify characteristics of the sources of evidence used as eligibility criteria (e.g., years considered, language, and publication status), and provide a rationale. | 5 |
| Information sources* | 7 | Describe all information sources in the search (e.g., databases with dates of coverage and contact with authors to identify additional sources), as well as the date the most recent search was executed. | 4-5 |
| Search | 8 | Present the full electronic search strategy for at least 1 database, including any limits used, such that it could be repeated. | Online Appendix 1 |
| Selection of sources of evidence† | 9 | State the process for selecting sources of evidence (i.e., screening and eligibility) included in the scoping review. | 5-6 |
| Data charting process‡ | 10 | Describe the methods of charting data from the included sources of evidence (e.g., calibrated forms or forms that have been tested by the team before their use, and whether data charting was done independently or in duplicate) and any processes for obtaining and confirming data from investigators. | 6 |
| Data items | 11 | List and define all variables for which data were sought and any assumptions and simplifications made. | 6 |
| Critical appraisal of individual sources of evidence§ | 12 | If done, provide a rationale for conducting a critical appraisal of included sources of evidence; describe the methods used and how this information was used in any data synthesis (if appropriate). | N/A |
| Synthesis of results | 13 | Describe the methods of handling and summarizing the data that were charted. | 6 |
| **RESULTS** | | | |
| Selection of sources of evidence | 14 | Give numbers of sources of evidence screened, assessed for eligibility, and included in the review, with reasons for exclusions at each stage, ideally using a flow diagram. | 7 |
| Characteristics of sources of evidence | 15 | For each source of evidence, present characteristics for which data were charted and provide the citations. | 7, Table 1 |
| Critical appraisal within sources of evidence | 16 | If done, present data on critical appraisal of included sources of evidence (see item 12). | N/A |
| Results of individual sources of evidence | 17 | For each included source of evidence, present the relevant data that were charted that relate to the review questions and objectives. | 8-10, Table 1, Table 2 |
| Synthesis of results | 18 | Summarize and/or present the charting results as they relate to the review questions and objectives. | 8-10 |
| **DISCUSSION** | | | |
| Summary of evidence | 19 | Summarize the main results (including an overview of concepts, themes, and types of evidence available), link to the review questions and objectives, and consider the relevance to key groups. | 17-23 |
| Limitations | 20 | Discuss the limitations of the scoping review process. | 22 |
| Conclusions | 21 | Provide a general interpretation of the results with respect to the review questions and objectives, as well as potential implications and/or next steps. | 23 |
| **FUNDING** | | | |
| Funding | 22 | Describe sources of funding for the included sources of evidence, as well as sources of funding for the scoping review. Describe the role of the funders of the scoping review. | 24 |

JBI = Joanna Briggs Institute; PRISMA-ScR = Preferred Reporting Items for Systematic reviews and Meta-Analyses extension for Scoping Reviews.

* Where *sources of evidence* (see second footnote) are compiled from, such as bibliographic databases, social media platforms, and Web sites.

† A more inclusive/heterogeneous term used to account for the different types of evidence or data sources (e.g., quantitative and/or qualitative research, expert opinion, and policy documents) that may be eligible in a scoping review as opposed to only studies. This is not to be confused with *information sources* (see first footnote).

‡ The frameworks by Arksey and O’Malley (6) and Levac and colleagues (7) and the JBI guidance (4, 5) refer to the process of data extraction in a scoping review as data charting*.*

§ The process of systematically examining research evidence to assess its validity, results, and relevance before using it to inform a decision. This term is used for items 12 and 19 instead of "risk of bias" (which is more applicable to systematic reviews of interventions) to include and acknowledge the various sources of evidence that may be used in a scoping review (e.g., quantitative and/or qualitative research, expert opinion, and policy document).

*From:* Tricco AC, Lillie E, Zarin W, O'Brien KK, Colquhoun H, Levac D, et al. PRISMA Extension for Scoping Reviews (PRISMAScR): Checklist and Explanation. Ann Intern Med. 2018;169:467–473. [doi: 10.7326/M18-0850](http://annals.org/aim/fullarticle/2700389/prisma-extension-scoping-reviews-prisma-scr-checklist-explanation).

**Online Appendix 3:** Risk of bias summary table for all included nonrandomized studies of interventions


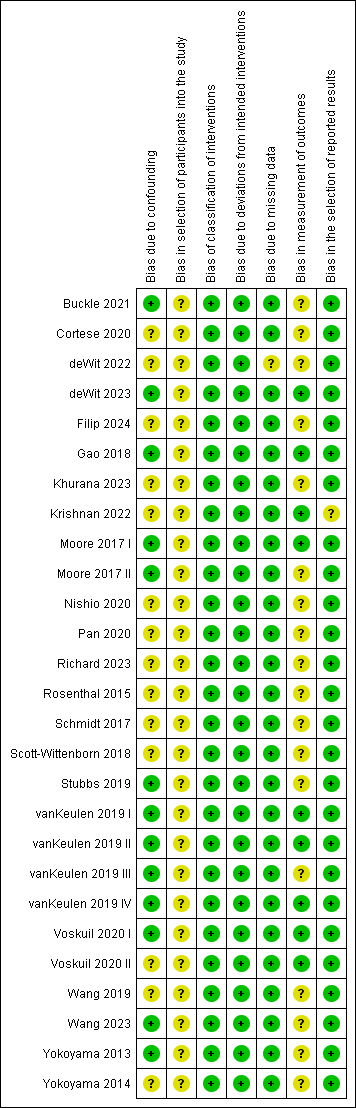


**Online Appendix 4**: Tumor sites of each study.

| **Study** | **Patients  (n)** | **Oropharynx*** | **Tonsil** | **Tongue*** | **Base of Tongue** | **Oral Tongue** | **Buccal** | **Oral Cavity (Floor of Mouth)*** | **Larynx** | **Nasal Cavity** | **Parotid** | **Lip** | **Maxillary Sinus** | **Other or Unspecified** |
| --- | --- | --- | --- | --- | --- | --- | --- | --- | --- | --- | --- | --- | --- | --- |
| Buckle 2021 | 10 |  |  | 10 |  |  |  |  |  |  |  |  |  |  |
| Cortese 2020 | 4 |  |  |  |  |  | 1 | 2 | 1 |  |  |  |  |  |
| de Wit 2022 | 16 |  |  | 12 |  |  | 1 | 3 |  |  |  |  |  |  |
| de Wit 2023 | 65 | 1 |  | 30 |  |  | 5 | 30 |  |  |  |  |  |  |
| Filip 2024 | 7 |  |  |  |  |  |  | 4 | 1 | 2 |  |  |  |  |
| Gao 2018 | 21 | 1 |  |  |  |  |  | 17 |  | 2 |  |  |  | 1 |
| Khurana 2023 | 18 |  | 8 |  | 2 |  |  |  |  |  |  |  |  |  |
| Krishnan 2022 | 18 |  |  | 8 |  |  | 2 | 8 |  |  |  |  |  |  |
| Moore 2017 I | 15 |  |  |  |  |  |  |  |  |  |  |  |  | 15 |
| Moore 2017 II | 6 |  |  |  |  |  |  | 4 |  |  |  |  |  | 2 |
| Nishio 2020 | 24 |  |  |  |  |  |  | 8 |  |  |  |  |  | 16 |
| Pan 2020 | 20 |  |  | 8 |  |  | 5 | 6 |  |  |  | 1 |  |  |
| Richard 2023 | 8 |  |  |  |  |  |  |  |  |  | 2 |  |  | 1 |
| Rosenthal 2015 | 12 | 1 |  |  |  |  |  | 7 |  | 1 |  | 1 |  | 2 |
| **Study** | **Patients  (n)** | **Oropharynx*** | **Tonsil** | **Tongue*** | **Base of Tongue** | **Oral Tongue** | **Buccal** | **Other Oral Cavity*** | **Larynx** | **Nasal Cavity** | **Parotid** | **Lip** | **Maxillary Sinus** | **Other or Unspecified** |
| Schmidt 2017 | 55 |  |  |  |  |  |  |  |  |  |  |  |  | 18 |
| Scott-Wittenborn 2018 | 6 |  | 3 |  | 1 |  |  |  |  |  |  |  |  | 2 |
| Stubbs 2019 | 14 | 3 | 5 |  | 3 |  |  |  |  |  | 3 |  |  |  |
| van Keulen 2019 I | 12 |  |  | 6 |  |  | 4 | 2 |  |  |  |  |  |  |
| van Keulen 2019 II | 14 |  |  | 4 |  |  | 2 | 4 |  |  |  |  | 1 |  |
| van Keulen 2019 III | 20 |  |  |  |  | 8 | 2 | 6 |  |  |  |  | 1 | 1 |
| van Keulen 2019 IV | 8 |  |  | 3 |  |  | 2 | 1 |  |  |  |  |  |  |
| Voskuil 2020 I | 15 |  |  |  |  |  |  |  |  |  |  |  |  | 15 |
| Voskuil 2020 II | 30 |  |  |  |  |  |  |  |  |  |  |  |  | 13 |
| Wang 2019 | 12 |  |  | 6 |  |  | 2 | 3 |  |  |  | 1 |  |  |
| Wang 2023 | 10 |  |  |  |  |  |  |  |  |  |  |  |  | 9 |
| Yokoyama 2013 | 9 | 2 |  |  |  |  |  | 2 |  |  |  |  |  | 1 |
| Yokoyama 2014 | 6 |  |  |  |  |  |  |  |  |  |  |  |  | 1 |
| Total | 455 | 8 | 16 | 87 | 6 | 8 | 26 | 107 | 2 | 5 | 5 | 3 | 2 | 97 |

* Includes other sites

Note: In certain studies, specific tumor site was not available for each patient.
